# P300 brain computer interface: current challenges and emerging trends

**DOI:** 10.3389/fneng.2012.00014

**Published:** 2012-07-17

**Authors:** Reza Fazel-Rezai, Brendan Z. Allison, Christoph Guger, Eric W. Sellers, Sonja C. Kleih, Andrea Kübler

**Affiliations:** ^1^Biomedical Signal and Image Processing Laboratory, Department of Electrical Engineering, University of North Dakota, Grand ForksND, USA; ^2^Cognitive Neuroscience Laboratory, Department of Cognitive Science, University of California at San Diego, La JollaCA, USA; ^3^g.tec Medical Engineering GmbH/Guger Technologies OGGraz, Austria; ^4^ETSU Brain-Computer Interface Laboratory, East Tennessee State University, Johnson CityTN, USA; ^5^Department of Psychology I, University of WürzburgWürzburg, Germany

**Keywords:** brain computer interface, P300, event-related potential

## Abstract

A brain-computer interface (BCI) enables communication without movement based on brain signals measured with electroencephalography (EEG). BCIs usually rely on one of three types of signals: the P300 and other components of the event-related potential (ERP), steady state visual evoked potential (SSVEP), or event related desynchronization (ERD). Although P300 BCIs were introduced over twenty years ago, the past few years have seen a strong increase in P300 BCI research. This closed-loop BCI approach relies on the P300 and other components of the ERP, based on an oddball paradigm presented to the subject. In this paper, we overview the current status of P300 BCI technology, and then discuss new directions: paradigms for eliciting P300s; signal processing methods; applications; and hybrid BCIs. We conclude that P300 BCIs are quite promising, as several emerging directions have not yet been fully explored and could lead to improvements in bit rate, reliability, usability, and flexibility.

## Introduction

Brain-computer interface (BCI) systems allow users to communicate without movement and provide a direct electronic interface to convey messages and commands from the brain to a computer (Wolpaw et al., 2002). A BCI system monitors conscious electrical brain activity via electroencephalogram (EEG) signals and detects patterns that are generated by the user. After the EEG is digitized, it is processed via digital signal processing algorithms to convert the EEG into a real-time control signal (Mason et al., 2007). By establishing a communication link between a subject and a computer, BCI can enable physically disabled people to perform many activities, which improve their quality of life and productivity, allowing them more independence (Wolpaw et al., 2002).

BCIs are named according to the type of brain activity used for control. Among several categories of EEG-based BCIs, including P300 (Farwell and Donchin, 1988), steady state visual evoked potential (SSVEP) (Herrmann, 2001), event related desynchronization (ERD) (Pfurtscheller and Neuper, 2001), and slow cortical potential based (Birbaumer et al., 2000), in this paper, only the P300-based BCI is reviewed. This type of BCI has recently been the focus of many studies, is relatively easy to use for a control signal, and has shown great potential to be used in several different applications.

The P300 is a component of the event-related potential (ERP) first reported by Sutton (Sutton et al., 1967). An ERP is an electrophysiological response to an internal or external stimulus. It is a fluctuation in the EEG that is elicited by and is time-locked to a sensory, motor, or cognitive event. The P300 is the largest ERP component and can be generated during an oddball paradigm. In an oddball paradigm, subject is presented with a sequence of events that can be categorized into two classes such that one of them is rarely presented (Donchin and Coles, 1988). The infrequent event generates the P300 peak about 300 ms after stimulus onset. P300 is involved with the process of memory modification or learning and things appear to be learned if, and only if, they are surprising (Donchin, 1981). In this paper, we discuss the progress in the P300 based BCIs, current challenges, and emerging trends.

The paper is organized as follows. The next section discusses the current status of P300 BCIs. In section “Visual P300 Paradigms,” several paradigms for eliciting the visual P300 are presented. Section “P300 Detection” discusses several P300 detection challenges and associated performance limitations of the P300 BCI. Several different applications of the P300 BCI that are being investigated and developed are discussed in section “Other P300-BCI Applications.” Finally, we end with concluding remarks in section “Validation.”

## Current status of P300 BCIs

Although the P300 BCI was introduced in 1988 (Farwell and Donchin, 1988), it initially received very little attention. From 1988 to 2000, there were no P300 BCI peer-reviewed papers (Donchin et al., 2000). The next five years saw only a modest increase in P300 BCI articles, which often relied on offline analyses, such as analyzing other groups' data from the 2003 BCI Data Analysis Competition (Allison and Pineda, 2003; Xu et al., 2004).

However, in the past few years, P300 BCIs have clearly emerged as one of the main BCI categories. P300 BCIs have consistently exhibited several appealing features—they are relatively fast, effective for most users, straightforward, and require practically no training. Recent work has shown that P300 BCIs can be used for a wide range of different functions and can work with disabled users in home settings (Kübler et al., 2009; Sellers et al., 2010; Kleih et al., 2012), although some concerns about gaze shifting have emerged (Allison et al., 2008; Brunner et al., 2010; Treder and Blankertz, 2010). In addition, new paradigms for eliciting the P300 have been introduced (Fazel-Rezai and Abhari, 2009; Townsend et al., 2010), and new ways to flash or otherwise change stimuli could enhance ERPs and improve classification (Kaufmann et al., 2011; Jin et al., 2012). Overall, it is likely that P300 BCIs will remain prominent in the foreseeable future, but will probably grow further and further away from the canonical 6 × 6 matrix with single row and column flashes described in the first two P300 BCI articles.

### P300 papers submitted in journals since 2000

**Figure 1:**
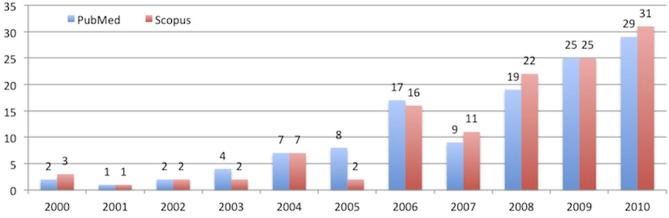
**Number of published journal papers in PubMed and Scopus from 2000 to 2010 when “[(P300 OR P3) AND (BCI OR Brain Computer Interface)]” keyword was used**.

The past few years have seen a strong increase in P300 BCI research. Figure 1 shows the number of peer-reviewed journal publications that were identified via PubMed and Scopus search engines from 2000 to 2010 with the phrase “[(P300 OR P3) AND (BCI OR Brain Computer Interface)].” Conference proceedings were removed from the search. These articles reflect numerous novel directions. In addition to improving information transfer rate, there has been considerable success extending P300 BCIs to new tasks, paradigms, and applications.

### 2010 BCI award submission statistics

To highlight trends and developments of BCI technology, g.tec (Medical Engineering GMBH, Austria) began to sponsor the Annual BCI Award in 2010. Fifty-seven projects were submitted to the BCI Award 2010 and an international committee nominated the 10 top-ranked candidates (Guger, 2011).

Table 1 categorizes the BCI Award 2010 nominees into utilized control signals and application areas. Of the eight nominated projects that used EEG as input signal, six utilized the N200/P300 response. N200 is a negative peak in the ERP that appears about 200 ms after a stimulus onset (Hong et al., 2009). The committees decision to select a number of P300 BCI paradigms is reflected by several reasons: (1) the P300 response is easy to measure and non-invasive, (2) it requires less than 10 min of training, (3) it works with the majority of subjects including those with the severe neurological disease, and (4) gives a goal-oriented control signal that is especially suited for spelling and control application where no continuous control signal is needed (e.g., Internet surfing, painting). All the spelling/Internet/art applications selected by the BCI Award committee were controlled with the P3 N200/P300 strategy. The two other projects used motor imagery (MI) to generate a continuous control signal. Both MI projects used the BCI system for the activation of the sensorimotor cortex for stroke rehabilitation, which is not possible with N200/P300- or SSVEP-based BCI systems. In the 2010 competition, 40.4% of 57 submissions used MI, 29.8% used P300 and 8.9% used the SSVEP principles, and the rest used other modalities.

**Table 1:** **Categorization of the 10 BCI Award nominees**.

**Title**	**Authors *Affiliations***	**Control signal**	**Application**
A high speed word spelling BCI system based on code modulated visual evoked potentials	Guangyu Bin, Xiaorong Gao, Shangkai Gao *Department of Electrical Engineering, Tsinghua University, Beijing, China*	N200 P300	Spelling Internet Art
Motor imagery-based Brain-Computer Interface robotic rehabilitation for stroke	Cuntai Guan, Kai Keng Ang, Kok Soon Phua, Chuanchu Wang, Zheng Yang Chin, Haihong Zhang, Rongsheng Lin, Karen Sui Geok Chua, Christopher Kuah, Beng Ti Ang *Institute for Infocomm Research, Agency for Science, Technology and Research, Singapore*	MI	Stroke
An active auditory BCI for intention expression in locked-in	Jing Guo, Shangkai Gao, Bo Hong *Department of Electrical Engineering, Tsinghua University, Beijing, China*	N200 P300	Spelling Internet Art
Brain-actuated Google search by using motion onset VEP	Tao Liu, Shangkai Gao, Bo Hong *Department of Electrical Engineering, Tsinghua University, Beijing, China*	N200 P300	Spelling Internet Art
Brain Painting—“Paint your way out”	Harry George, Sebastian Halder, Adi Hösle, Jana Münßinger, Andrea Kübler *Department of Psychology I, University of Würzburg, Würzburg, Germany*	N200 P300	Spelling Internet Art
Thought Recognition with Semantic Output Codes	Mark Palatucci, Dean Pomerleau, Geoff Hinton, Tom Mitchell *Brain-Computer Interface Research Center for Translational Neurological Research, Wadsworth Center, New York State Department of Health, Albany, New York*	fMRI	Spelling Internet Art
Predictive Spelling with a P300-based BCI: Increasing Communication Rate	David B. Ryan and Eric W. Sellers *ETSU Brain-Computer Interface Laboratory, East Tennessee State University, Johnson City, TN, USA*	N200 P300	Spelling Internet Art
Innovations in P300-based BCI Stimulus Presentation Methods	George Townsend *ETSU Brain-Computer Interface Laboratory, East Tennessee State University, Johnson City, TN, USA*	N200 P300	Spelling Internet Art
Operant conditioning to identify independent, volitionally-controllable patterns of neural activity	Steven M. Chase, Andrew S. Whitford, Andrew B. Schwartz *Department of Neurobiology, University of Pittsburgh*	Spikes	Algorithm Development
Neurorehabilitation for Chronic-Phase Stroke using a Brain-Machine Interface	Kimiko Kawashima, Keiichiro Shindo, Junichi Ushiba, Meigen Liu *Keio University, Tokyo, Japan*	MI	Stroke

## Visual P300 paradigms

One of the most important facets of a P300 BCI involves eliciting large differences between target and non-target ERPs. Typically, a visual paradigm is displayed on a computer screen for stimulation. For a long time, the row/column (RC) paradigm originally introduced by Farwell and Donchin in 1988 (Farwell and Donchin, 1988) was the most common paradigm for P300 BCI. Recently, there have been several attempts to move beyond this paradigm. In this section, we review the original RC paradigm and its modifications and discuss a few paradigms for better visual feedback in a closed loop P300 BCI system.

### Row/column and its modifications

**Figure 2:**
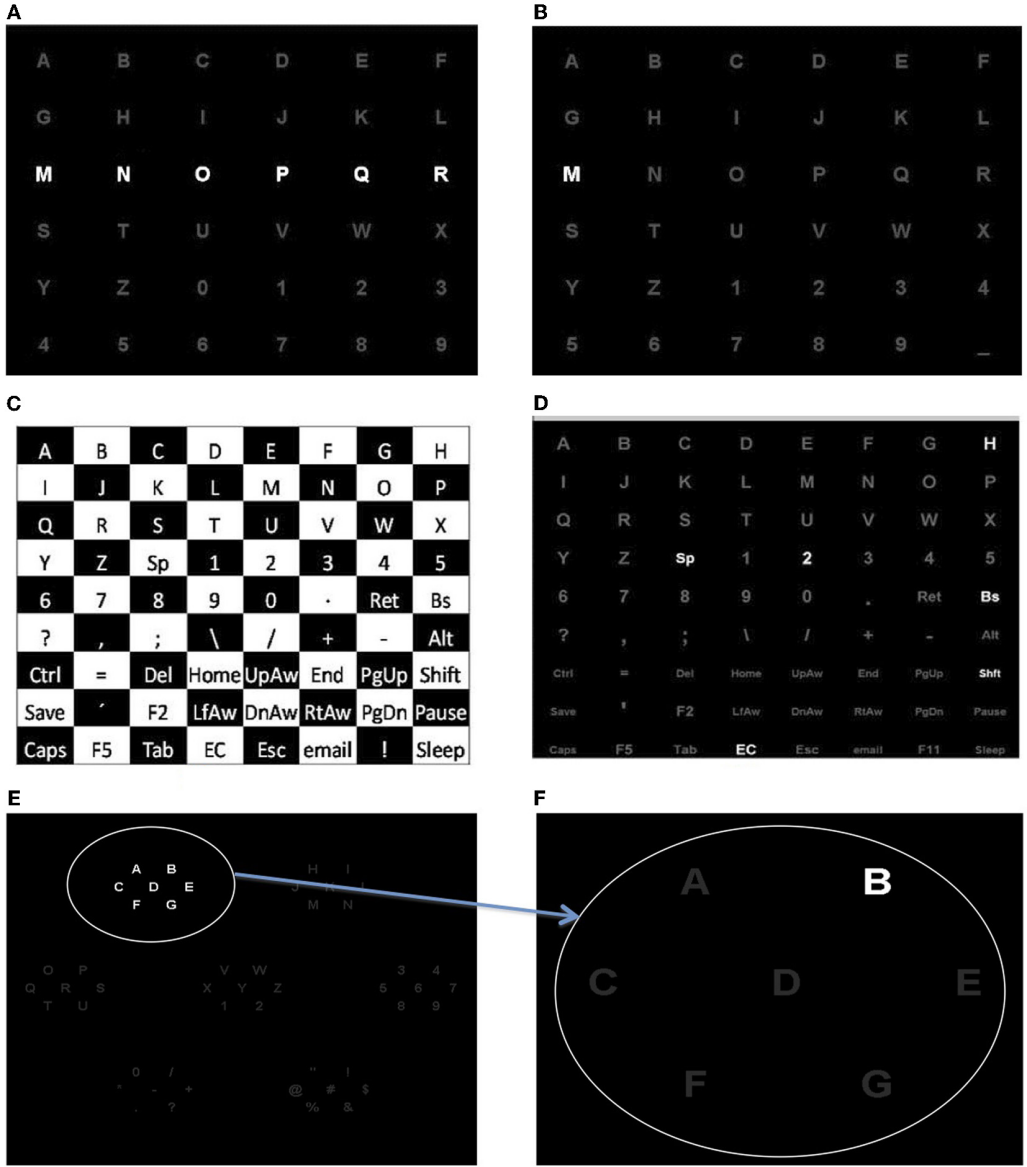
**(A)** Row/column paradigm: row and columns are flashed. **(B)** Single character paradigm: each character is flashed. **(C,D)** Checkerboard paradigm. **(E,F)** Region based paradigm where a set of characters in level 1 **(E)** are expanded in level 2 for spelling character “B” **(F)**.

The Farwell and Donchin (Farwell and Donchin, 1988) speller BCI is shown in Figure 2. In this system, a 6 × 6 matrix of symbols, comprising all 26 letters of the alphabet and 10 digits (0–9), is presented to the user on a computer screen and each row and column are flashed in a random order (Figure 2A). At any given moment, the user focuses on the character he or she wishes to communicate and silently counts the number of times the desired character flashes. In response to flashing of the desired character, the row and column of the desired character elicit a P300, whereas the other 10 rows and columns do not. Detection of the P300 makes it possible to match the responses to one of the rows and one of the columns, and thus to identify the desired character.

The P300 speller paradigm has been a benchmark for P300 BCI systems. One of the greatest advantages of the P300 BCI is that it does not require intensive user training, as the P300 component results from endogenous attention-based brain function. However, P300 detection for real-time applications presents several challenges. Several issues need to be addressed before any P300-based BCI can be taken outside the research laboratory and put to practical use. Some important issues are that EEG signal patterns change in response to factors such as motivation, level of attention, fatigue, mental state, learning, and other non-stationarities that exist in the brain (Wolpaw et al., 2002). In addition, users have unique EEG patterns that make it necessary for individualized calibration. These factors create a need for advanced digital signal processing algorithms to detect the P300 accurately and quickly. In spite of all the advanced signal processing algorithms applied to this paradigm, its use in real-world applications has been limited. One of the obstacles has been low real-time P300 detection accuracy. Several human perceptual phenomena such as attentional blink, repetition blindness, and habituation are potential sources of error in P300 detection and have been addressed in the literature (Fazel-Rezai, 2007, 2009; Citi et al., 2008; Jin et al., 2010; Townsend et al., 2010).

Recently, two studies demonstrated that the matrix speller can be operated more efficiently when target symbols are fixated (Brunner et al., 2010; Treder and Blankertz, 2010). To alleviate this, Treder (Treder and Blankertz, 2010) performed an online study using three different variants of fast-paced, non-gaze-dependent visual spellers. Participants could use covert spatial attention, non-spatial feature attention (i.e., attention to color and form) in two paradigms and in a third paradigm they could use covert, feature, and overt attention. They achieved mean symbol selection accuracies of roughly 85–90% for one out of thirty symbols and demonstrated that overt attention is not necessary for highly accurate responses. Other work explored different matrix sizes (Allison and Pineda, 2003; Sellers et al., 2006) and investigated replacing the elements of the matrix with icons or other choices instead of single letters and numbers (Bayliss and Ballard, 2000; Serby et al., 2005; Allison and Pineda, 2006; Bell et al., 2008; Salvaris and Sepulveda, 2009). In addition to these efforts focused on improving P300 BCI performance or allowing new paradigms for communication, newer work has addressed usability and other new paradigms. In the following sections a few recently introduced paradigms are explained.

### Single character

The single character (SC) speller randomly flashes one character at a time with a brief delay between flashes (Figure 2B). The SC speller has a longer delay between flashes than the RC and character classification can be made with fewer flashes per character. With a 60 ms flash and 40 ms between flashes, 54 s are needed to flash each character 15 times if a 36 character matrix is used. In contrast with a 100 ms flash and 60 ms between flashes, the RC requires only 28.8 s to present 30 flashes of each character. Accordingly the RC flasher is about two times faster than the SC flasher. However, the SC speller (15 flashes) results in larger P300 amplitudes compared to the RC speller with 15 flashes per column and row (Guger et al., 2009). Guger et al. (Guger et al., 2009) compared the SC and RC speller in a study where five characters were spelled. RC subjects reached a mean accuracy of 85.3% and SC subjects reached 77.9% (*N* = 19).

### Checkerboard

The checkerboard (CB) paradigm was designed to overcome two specific problems with the standard RC presentation method. One, the CB eliminates instances when the same character flashes twice in succession (also called a double target item flash). Two, the CB paradigm reduces the amount of distraction and/or inherent noise (i.e., non-target items receiving apparent target responses) to the RC paradigm. Townsend et al. (Townsend et al., 2010) showed that the CB produced significant improvements in accuracy, as compared to the RC paradigm. The CB paradigm disassociates the rows and columns of the matrix, thereby eliminating double flashes and significantly reducing distraction (Townsend et al., 2010). The original CB paradigm presented stimuli in an 8 × 9 matrix as shown in Figures 2C and D. A virtual CB is superimposed onto the 72 items contained in the matrix (not seen by viewers). Items in “white” squares randomly populate one 6 × 6 matrix and items in “black” squares randomly populate a second 6 × 6 matrix. Simultaneous adjacent flashes are prohibited by the segregation of adjacent items into separate flash groups, whereas the presentation of the 8 × 9 matrix appears to flash at random. Once the randomly populated virtual rows and columns are filled, the characters flash in sequential order: first, the rows of the white matrix; second, the rows of the black matrix; third, the columns of the white matrix; fourth, the columns of the black matrix are flashed. Thus, the paradigm places a minimum of six-six item group flashes or a maximum of 18-six items group flashes between the flashes of any given matrix item. After all rows and columns in both matrices have been flashed (24 flashes, comprising one complete sequence), the positions of the characters in each virtual matrix are re-randomized and the next sequence of flashes begins.

It is well documented that flanker tasks significantly increase reaction time when nearby items belong to a response class that competes with the target class (Sanders and Lamers, 2002). In the RC paradigm, when adjacency-distraction occurs, incorrect selections are typically in the same row or column as the desired target (Donchin et al., 2000; Fazel-Rezai, 2007; Townsend et al., 2010).

Imposing the constraint of a minimum of six intervening flashes avoids the problem of overlapping target epochs (Martens et al., 2009). The expansion to an 8 × 9 matrix increases the amplitude of target items by reducing the probability of the target stimulus occurring (Allison and Pineda, 2003; Sellers et al., 2006). Follow-up studies with the CBP have already begun to improve on the original design. In one experiment, a predictive spelling application was added to the CB paradigm and increased effective information transfer rate (Ryan et al., 2011). Another study included a mindfulness induction to evaluate the effects of enhanced attentional resources, showing that mindfulness induction significantly improved classification accuracy over a non-induction control group in the RC and CB paradigms (Lakey et al., 2011). A third study demonstrated that CB paradigm could be further improved by suppressing (i.e., not flashing) the items surrounding the attended item during calibration. As compared to the standard CB paradigm calibration procedure, subsequent online results showed that the suppression calibration procedure produced significantly better performance in the testing phase of the experiment. In the testing phase of the experiment, items surrounding the target item flashed. Additionally, identical randomly generated sequences for the classifier derived from suppression calibration and those derived from non-suppression calibration.

### Region-based

The idea of region-based (RB) paradigm (Fazel-Rezai and Abhari, 2009) is to have flashes of several regions instead of rows and columns. The character recognition is performed in two levels (Fazel-Rezai and Ahmad, 2011). In the first level, the characters are placed into seven groups located at different regions of the screen, as shown in Figure 2E. Similar to the Farwell and Donchin paradigm, the user is instructed to attend a specific character in one of the seven groups while each group of seven characters randomly flashes. After several flashes of each group the desired group is identified. In the second level, individual characters of the selected group are singly distributed into the seven regions as shown in Figure 2F. Similarly to the first level, different regions are flashed while the subject attends to one region (i.e., character). The desired character is selected by identifying one of the seven regions.

It was shown (Fazel-Rezai and Abhari, 2009; Fazel-Rezai and Ahmad, 2011) that the RB paradigm decreased the near-target effect and human error and adjacency problem significantly (Fazel-Rezai, 2007). It was found that the overall spelling accuracies averaged for the same set of subjects, trials, and characters for RC, SC, and two variations of RB paradigms were 85%, 72.2%, 90.6%, and 86.1%, respectively, (Fazel-Rezai et al., 2011). The RB and CB paradigms show a new direction in P300 BCI that can produce superior performance, and may generally reduce the need for the RC approach to P300 BCIs.

### Moving and alternative stimuli

Another emerging approach involves motion- rather than flashing stimuli. Guo (Guo et al., 2008) introduced a new paradigm with five possible targets that represented left, right, up, down, and enter commands for a virtual keyboard. In this task, instead of flashing, a vertical bar below each of the five stimuli appeared and moved leftward at random intervals for 140 ms. Results from offline analyses suggested that this approach yielded visual evoked potentials that could be useful in an offline BCI. The waveforms did show a difference in P3 activity, but the authors suggested that their new approach could be advantageous by highlighting earlier components such as N2, which is recorded from occipital-parietal regions and is related to area V5 of the visual cortex.

Additional work has explored the moving stimulus paradigm. Hong (Hong et al., 2009) described an offline comparison of two 6 × 6 matrices: one based on conventional flashing stimuli, and another based on moving vertical bars. Results showed that the moving stimuli elicited a stronger early negative component than the flashing stimuli. Liu (Liu et al., 2010) presented the first online BCI based on this new approach. After an offline calibration session, subjects participated in online sessions that evaluated a generic system to select one of six letters and a tool for web searching. An information transfer rate of 42.1 bits/min was achieved, averaged over 12 subjects.

Jin et al. (2012) compared three BCIs: one based on conventional flashing stimuli, another based on moving stimuli, and a hybrid condition in which the stimuli both flashed and moved. All 10 subjects could use the new hybrid BCI system. Results also showed that the hybrid approach was statistically superior to the other two approaches in accuracy and practical bit rate.

Familiar faces, which are known to elicit the N170 and N400f (“f” for faces) components of the ERP (Eimer, 2000), have also been used in a P300 BCI (Kaufmann et al., 2011). Letters of the P300 matrix were superimposed on the flashed behind the letters. Taking into account the additional ERPs, the number of sequences necessary to achieve 100% accuracy could be significantly decreased and thus, bitrate increased.

The initial studies examining moving stimuli suggest that the canonical “flash” approach used in most P300 BCIs may not be as good as other stimulation methods. Just as future SSVEP BCIs might more frequently employ newer stimulation approaches (Bin et al., 2009, 2011), P300 BCIs might also start using stimuli that move or otherwise change in ways that elicit more distinct ERPs. It is likely that paradigms such as the moving stimuli will be further optimized in a fashion similar to the flashing BCIs because so many possible stimulus manipulations exist.

## P300 detection

To properly detect P300 and increase transfer rate and accuracy, which are interdependent, several issues should be considered. (1) Attentional blink: it occurs if the intervals between two targets are less than 500 ms (Raymond et al., 1992). (2) Repetition blindness: if two identical targets in a stream of non-targets are flashed at intervals between 100 to 500 ms, the second target may be missed (Kanwisher, 1987). (3) Target to target interval: P300 amplitude is related to the interval between target events (Gonsalvez and Polich, 2002). (4) Habituation: P300 amplitude often decreases with repeated presentation of the same stimulus although this may not occur in some BCI paradigms (Sellers et al., 2006). When the user loses focus on the target character, the P300 is not elicited, and thus accurate classification is not possible. In addition, human error affects the accuracy of the P300 BCI (Fazel-Rezai, 2007), which should be considered in the design of paradigms. Motivation may also influence performance in BCIs (Kleih et al., 2010).

Similar to any pattern recognition problem, the P300 detection requires preprocessing, feature extraction, and classification. The first step is to remove noise through preprocessing. Bandpass filtering is a common preprocessing method in P300 speller paradigms. Typically, raw EEG signals are filtered with a digital bandpass filter with a low cutoff frequency of 0.1 Hz and high cutoff frequency of 30 Hz. Traditionally, ERPs are averaged to enhance P300 amplitude and suppress background EEG activity. Then, features should be extracted from EEG signals for P300 detection. Different methods have been used for this purpose such as discrete wavelet transform (Donchin et al., 2000), independent component analysis (Xu et al., 2004; Serby et al., 2005), and principal component analysis (McGillem and Aunon, 1977). The final step is classification. Farwell and Donchin used step-wise discriminant analysis (SWDA) followed by peak picking and covariance evaluation (Farwell and Donchin, 1988). Other methods have also been used for the P300 detection such as, support vector machine (SVM) (Thulasidas et al., 2006), and linear discriminant analysis (LDA) (Guger et al., 2009). Although different features and classifiers have been compared (Mirghasemi et al., 2006a,b), there has not been a comprehensive comparison of all different features extraction and classification methods applied to the same data set. One study has, however, examined this issue. Krusienski et al. (2006) showed that SWDA and Fisher's linear discriminant (FLD) provided the best overall performance and implementation characteristics for practical classification, as compared to Pearson's correlation method (PCM), a linear support vector machine (LSVM), and a Gaussian kernel support vector machine (GSVM). This indicates that the error, mostly due to lack of P300 in EEG, resulted from factors such as human error, adjacency effect, or mental fatigue explained in the previous sections.

As an illustrative example, P300 detection process in the RC speller is explained in this section. Rows and columns are highlighted randomly. The system sends an ID of the flashing character to the signal processing. The signal processing generates a buffer for each character and stores the incoming EEG data around the flash. This is done until all RC buffers are filled with X epochs (e.g., 30 flashes of each character). Then feature extraction is performed. Finally, a classifier, such as LDA, creates classification coefficients that are applied to the EEG features to identify the matrix location most likely to be the desired character. Each feature selected by the LDA receives a weight that corresponds to the importance of the particular feature; features that account for more variance in the signal have larger values than features that account for less signal variance. If the LDA classifier is correct, the character that the subject intended to select is presented on the computer screen as feedback to the subject. Then the process starts again with the next character.

## Other P300-BCI applications

There has been progress in developing BCI applications to accomplish new tasks or goals or explored to move a cursor in one of four directions instead of directly select items (Piccione et al., 2006; Citi et al., 2008). Another recent paper described a P300 BCI to control a mobile robot (Bell et al., 2008) or to control a wheelchair (Iturrate et al., 2009).

### Brain painting

An even more novel direction involves utilization of BCIs for creative expression and entertainment. One of the drawbacks to the majority of today's BCI systems has been their emphasis on function instead of usability, resulting in BCI software which lacks the appeal and user experience we have grown accustomed to from commercial products (Allison, 2009). Hence, BCIs might work, but are often confusing, counter intuitive, or boring for the user. When designing novel BCI systems, developers should consider signal acquisition methods, innovative paradigms, and also engaging interaction for the user. New interaction aspects have generally received less attention in BCI research.

BCIs that facilitate the completion of tasks that seem more natural or intuitive have been shown to yield numerous benefits. Development of the P300 BCI application known as “Brain Painting” (BP) was created with a “user-centered design” in mind (Kübler et al., 2008; Muenssinger et al., 2010).

**Figure 3:**
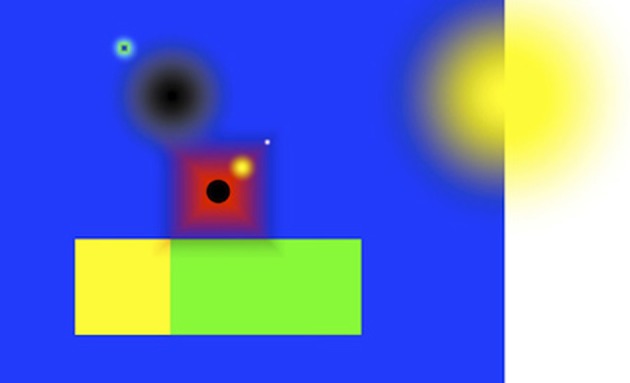
**Example of a brain painting picture painted by a healthy volunteer**.

By analyzing the wishes of end users, e.g., patients diagnosed with amyotrophic lateral sclerosis (ALS), the BP application fulfils basic human requirements of assisting expression, albeit through an “alternative communication channel.” In this case, the alternative channel is a creative means of picture drawing as shown in Figure 3. This has resulted in not only a new BCI design in terms of novelty, but one that improves mood, motivation, and quality of life in patient users. The positive emotions exhibited during creative and playful expression have been well documented in helping with patient rehabilitation (Radtke, 1994).

Users diagnosed with ALS recorded accuracy comparable or better than healthy controls and expressed high motivation, exceeding 8.5 on a visual analogue scale (VAS) ranging from 1 to 10 before and after painting sessions (Muenssinger et al., 2010). Accuracy of the P300 black and white BP matrix equaled that achieved using the P300-Spelling application (spelling: 93.20%, SD 7:5); painting: 92.60%, SD 5:7). BCI sessions lasted on average upwards of 1.5–3.5 h with repeated desire to re-use the system.

In comparison to the current P300 BCI applications, such as the speller, BP encourages improved user immersion with the BCI device. Critically, multiple extended sessions assists in further training the user with the P300 paradigm and results in improved accuracy as well as greater overall enjoyment of BCI use, which certainly is an advantage when trying to embed a new technology for widespread use.

### Virtual reality

**Figure 4:**
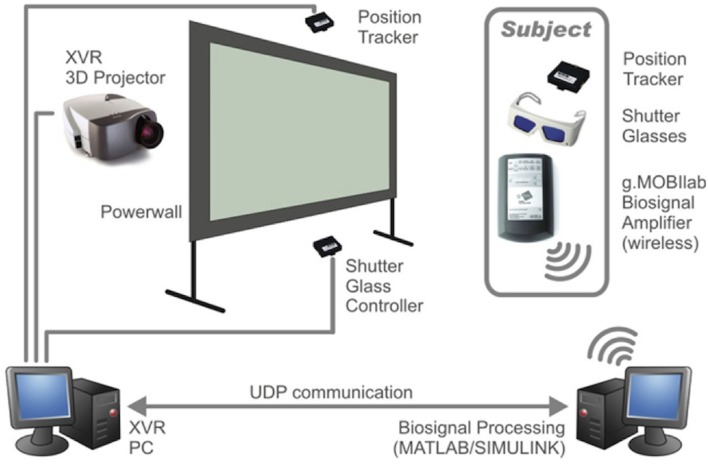
**Scheme of virtual environment setup**.

To operate a BCI to control a virtual environment, several demands must be met: (1) biosignal amplifiers must be functional while the subject is moving; (2) the recordings should ideally be done with a rather small portable device to avoid collisions and irritations within the environment; (3) the BCI system must be coupled with the virtual reality (VR) system for real-time experiments and (4) a special BCI communication interface must be developed to have enough degrees of freedom available to control the VR system. Figure 4 illustrates the necessary components in detail. A 3D projector is located next to a projection wall for back projections. The subject can be positioned in front the projection wall to avoid shadows and is equipped with position tracker to capture movements, shutter glasses for 3D effects and the biosignal amplifier including electrodes for EEG recordings. The XVR (eXtreme VR, VRmedia, Pisa, Italy) PC controls the projector, the position tracker, and the shutter glass. The biosignal amplifier is transmitting the EEG data to the SSVEP—P300 BCI system which is connected to the XVR PC via UDP connection to exchange control commands.

**Figure 5:**
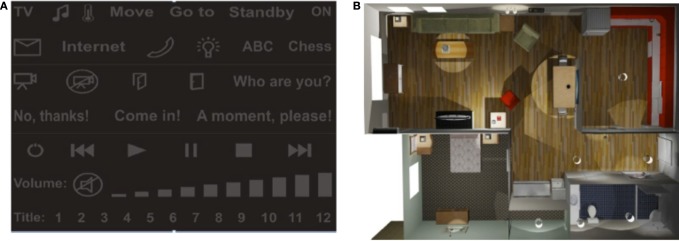
**(A)** Smart home interface mask. **(B)** Bird′s eye view of the virtual apartment with domotic devices to be operated like the TV set, music set, room light, or chess board.

The virtual smart home itself consists of different rooms whereby each room is equipped with several different devices that can be controlled: TV, MP3 player, telephone, lights, doors, etc. Therefore, all the different commands were summarized in seven control masks: a light mask, a music mask, a phone mask, a temperature mask, a TV mask, a move mask, and a go to mask. Figure 5 shows the light mask and as an example the corresponding XVR bird's eye view. For further details see (Edlinger et al., 2009).

### Gaming

Twitter (Twitter Inc.) is a social network that enables the user to send and read messages. The messages are limited to 140 characters and are displayed in the authors profile page. Messages can be sent via the Twitter website or via smart phones or SMS (Short Message Service). Twitter also provides an application programming interface to send and receive SMS. Second Life (SL) is another application which is a free 3D online virtual world developed by the American company Linden Lab. It was launched on June 23, 2003 and already five years later the platform had 15 million registered accounts whereas on average 60,000 users were online at the same time. Only the free client software package “Second Life Viewer” and an account are necessary to participate.

One of the main activities in SL is socializing with other so-called residents whereas every resident represents a person in the real world. Users can perform different actions like holding business meetings, take pictures or make movies, attend courses, etc. Communication takes place via text chats, voice chats, and gestures. Hence, handicapped people could also participate in SL like any other user if an appropriate interface were available. A P300 controller was interfaced using a SL controller in C++ Simulink S-functions.

**Figure 6:**
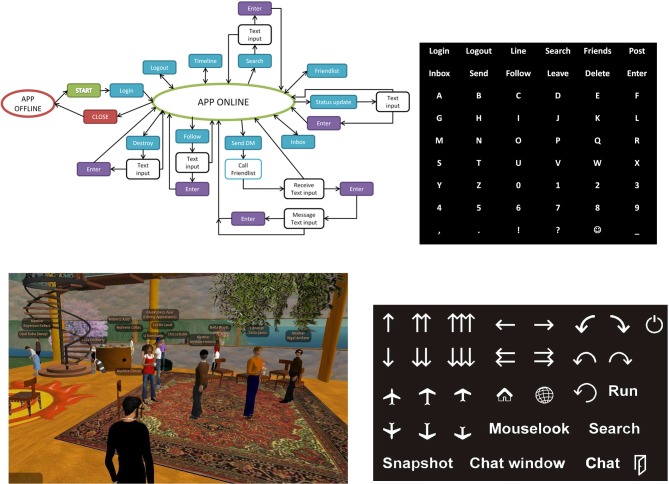
**Upper panel: UML diagram of service Twitter and P300—Twitter interface mask for control.** Lower panel: Screenshot of Second life situation and Second Life interface main mask to walk forward/backward, turn left/right, slide left/right, climb, teleport home, show map, turn around, activate/deactivate running mode, start/stop flying, decline, activate/deactivate mouse-look view, enter search mask, take snapshot, start chat, quit, and stand-by.

In order to participate in sending tweets and SL, appropriate interface masks were designed and the P300 base system was modified accordingly. The upper panel in Figure 6 shows an UML diagram of the actions required to use e.g., the service Twitter. Hence the standard P300 spelling matrix based on a 6 × 6 characters matrix was enhanced to provide the necessary commands. Therefore the first two lines contain the symbols representing corresponding Twitter services and the remaining characters are used for spelling purpose. The matrix contains a total of 54 characters. Initial training of the system was done for 10 characters. Then, another user asked questions via Twitter and the BCI User had to answer different questions every other day. Therefore, in total, the BCI User had to use the interface on nine different days and selected between 6 and 36 characters every day. The changes between the first and last sessions are noteworthy. The first session lasted 11:09 min and the user spelled 13 characters, but made three mistakes. The user was told to correct any mistake, which yielded an average of 51 s selection time per character. During the last session, the user spelled 27 characters in 6:38 min with only one mistake and an average selection time of 15 s per character. Also, the number of flashes per character was reduced from 8 to only 3 flashes to increase the speed (Edlinger and Guger, 2011).

For the control of SL, like the virtual smart home control, three different interface masks were developed. The lower panel in Figure 6 displays a screenshot of a SL scene and the main mask as shown having 31 different classes to select from. Other masks for control like “chatting” (55 classes) and “searching” (40 classes) were developed. Each of the icons represents an actual command associated with it. If a certain icon is selected, SL is notified to execute this individual action with actually using keyboard events. The SL control and performance have been tested in initial efforts, and preliminary results indicate performance very similar to the virtual smart home scenario (Edlinger and Guger, 2011).

### The first commercial BCI system

**Figure 7:**
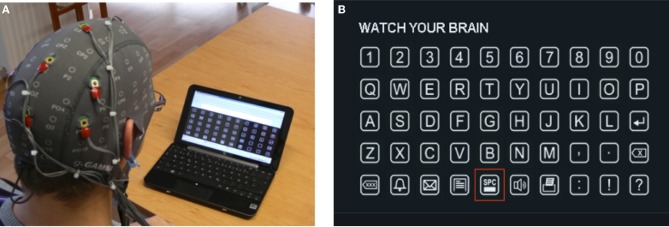
**(A)** The intendix BCI running on the laptop and user wearing the active electrodes. **(B)** User interface with 50 characters and computer keyboard like layout.

The Intendix BCI system was designed to be operated by caregivers or the patient's family at home. It consists of active EEG electrodes to avoid abrasion of the skin, a portable biosignal amplifier and a laptop or netbook running the software under Windows (Figure 7A). The electrodes are integrated into the cap to allow a fast and easy equipment mounting. The system allows viewing the raw EEG to inspect data quality, but automatically informs inexperienced users about the data quality on a specific channel.

This control can be realized by extracting the P300 evoked potential from the EEG data in real-time. Therefore, the characters of the English alphabet, Arabic numbers, and icons were arranged in a matrix on a computer screen (Figure 7B). Then the characters are highlighted in a random order and the person has the task to concentrate on the specific character he/she wants to spell. At the beginning, the BCI system is trained based on the P300 response of several characters with multiple flashes per character to adapt to the specific person.

When the system is started for the first time, user training has to be performed. Typically, the user has to spell 5–10 given target characters for training. The EEG data is used to calculate the user specific weight vectors, which is stored for later usage. Then the software switches automatically into the spelling mode and the user can spell as many characters as wanted.

The user can perform different actions: (1) copy the spelled text into an Editor, (2) copy the text into an email, (3) send the text via text-to-speech facilities to the loud speakers, (4) print the text, or (5) send the text via UDP to another computer. Specific icons exist for each of these functions.

The number of flashes for each classification can be selected by the user to improve speed and accuracy, or the user can also use a statistical approach that automatically configures the BCI accordingly. In the latter approach, no characters are selected if the user is not looking at the matrix or does not want to use the speller.

### P300 hybrid BCIs

Another emerging direction with P300 BCIs, and indeed all major BCI approaches, involves combining the BCI with another communication system. Some groups have begun developing “hybrid” BCI systems (Pfurtscheller et al., 2010; Allison et al., 2012), which may combine a P300 BCI with another BCI.

Combining a P300 BCI with an SSVEP BCI was first described two years ago (Allison et al., 2010). The proposed approach would involve letters or backgrounds that oscillate as well as flash, which may produce SSVEP activity that could help to confirm or identify the desired target. A somewhat different approach has recently been implemented in a P300/SSVEP hybrid BCI. In this approach, SSVEP activity is used to assess whether the subject is focused on the spelling task. If no SSVEP activity is found, then the system assumes that the user is not paying attention to the spelling system and does not output any characters (Panicker et al., 2011). The paradigm produces what could be considered a “no-control state” that is essential for practical BCI applications (e.g., Internet browsing, pauses to think about what to do next).

Recently, for the control of a smart home environment, a P300 based system was developed to selected different actions like switching on the TV. This BCI system was combined with an SSVEP system to switch on/off the flashing matrix (Edlinger et al., 2011). Other work combined a P300 BCI with a BCI based on imagined movement (Li et al., 2010). Six subjects viewed a display with several flashing boxes that contained the word “up,” “down,” or “stop.” By focusing on one of these boxes, subjects generated P300s that controlled the vertical position of a cursor. Subjects could simultaneously and independently control the horizontal position by imagining left or right hand movement.

A new hybrid BCI combines P300 and MI based BCIs for different processes (Su et al., 2011). Users can navigate continuously through a virtual Smart Home environment by imagining left or right hand movement. If the user entered one area of this virtual environment, a control panel appeared that allowed the subject to select an item on a television control panel. If the user entered a different area, then he or she saw a similar control panel for a stereo. Each control panel allowed the user to choose one of five items. Hence, this hybrid BCI allowed users to select the type of brain activity best suited to each task—imagined movement ERD for navigation and P300 for selection.

## Validation

One important emerging direction is field (i.e., external) validation with end users, particularly people with severe disabilities. Many groups, videos, and demonstrations only report performance with one user, or a selected group of users. Many reports only describe results in laboratory settings under ideal circumstances, and do not assess user preferences. Recent work has helped to counteract these problems. This section reviews results of a large scale field study with 100 healthy subjects, and other validations efforts with severely disabled users in home settings.

### Performance of the P300 speller in a group study

One hundred subjects tested a P300 based BCI system to spell a five character word with only 5 min of training. EEG data were acquired while the subject attended to a 36 character matrix to spell the word WATER. Two different versions of the P300 speller were used: (1) the RC speller that flashes an entire column or row of characters and (2) a SC speller that flashes each character individually. The subjects were free to decide which version to test. Nineteen subjects opted to test both versions The BCI system classifier was trained on the data collected for the word WATER. During the real-time phase of the experiment, the subject spelled the word LUCAS, and was provided with feedback as to the character selected by the classifier after each of the five letters. Close to seventy-three percent of subjects (72.8%; *N* = 81) were able to spell with 100% accuracy in the RC paradigm and 55.3% (*N* = 38) spelled with 100% accuracy in the SC paradigm. Less than 3% of the subjects did not spell any character correctly. This study shows that high spelling accuracy can be achieved with the P300 BCI system using approximately 5 min of training data for a large number of healthy subjects, and that the RC paradigm is superior to the SC paradigm. Eighty-nine percent of the 63 RC subjects were able to spell with accuracy 80–100%. A similar study using a MI BCI with *N* = 99 subjects showed that only 19% of the subjects were able to achieve an accuracy of 80–100% (Guger et al., 2003). This study was done with only two bipolar recordings to minimize the needed electrodes. But still these large differences in accuracy suggest that, even with much less training data, the P300 based BCI is superior to the MI BCI.

Very recent work repeated the same P300 training and spelling task with dry electrodes. Twenty-three subjects donned a dry electrode cap, trained the system with the word “LUCAS” and then spelled the word “WATER” with an intendiX P300 BCI. The subjects attained a mean accuracy of 90.4%, which was not statistically different from the mean accuracy of 91.0% reported in Guger et al. (2009). Since dry electrodes could substantially reduce preparation and cleaning time and dependence on outside support, this new work could help make P300 BCIs practical for more users (Guger et al., 2012).

These large differences in accuracy underscore why P300 BCIs may be appealing for certain tasks, such as direct selection of one out of several items. On the other hand, MI or SSVEP BCIs may be inherently better suited to continuous movement control, as discussed below (Jackson et al., 2006; Mason et al., 2007).

Overall, these group results are encouraging. Most users could choose one of 36 items with good accuracy, in a field setting with minimal training. Users generally reported via questionnaires that the system was not fatiguing or difficult. However, further research should compare different types of BCI approaches (P300, SSVEP, ERD, and others), possibly including non-EEG signals and/or hybrid combinations, across different applications to help identify the best BCI for each user. Different user groups should be considered, especially persons with neurological disorders and severe movement disabilities.

### Patient validation

Several studies have shown that P300 BCIs can provide communication to patients in field settings. The BP and Intendix systems described above were validated with ALS users in their home settings, as were other P300 BCI systems (Sellers et al., 2006, 2010; Vaughan et al., 2006; Nijboer et al., 2008). A recent conference presentation showed initial results of the first large scale field P300 BCI validation effort with patients in home settings. The results further indicated that P300 BCIs can be viable real-world communication systems for severely disabled users (Vaughan et al., 2006).

In a first systematic evaluation, Zickler (Zickler et al., 2011) had four severely disabled patients rate their subjective workload and satisfaction with a P300 based communication device which was integrated into a commercially available assistive communication Software (Qualilife®, 2012). Spelling, emailing, and internet browsing were with this P300 BCI application. All participants reported mental or temporal demand as the main source of their subjectively felt workload while physical demand, frustration and effort were generally judged as not contributing to subjectively felt workload. Users were very satisfied with the reliability and learnability of the BCI, but not with speed and aesthetic design (Zickler et al., 2011). Patients expressed that the “adjustment” (the gel and the relatively long preparation time) and “comfort” of the cap must be improved. They also expressed that the system be more compact and faster. Moreover, in its current state they would not only use the BCI if necessitated by disease progression. One person who used the BP BCI was an artist and reported that she had been unable to paint for years and the BCI allowed her to regain the ability to paint (Muenssinger et al., 2010). A person using a P300 BCI reported that he would be unable to work without the BCI (Sellers et al., 2010).

### Challenges and solutions

These results show that P300 BCI systems can provide effective communication. However, very few severely disabled users rely on them, or have been exposed to them. Several practical concerns limit wider P300 BCI adoption:

1. P300 BCIs, like any BCI, require significant support. An expert is needed to identify and assemble the components, customize parameters to each user, and address acute problems. Severely disabled users also need help to don the electrode cap before each session, and need someone to wash their hair and the cap afterward. Improved software and dry electrodes could help considerably (Guger et al., 2012).
2. Many P300 BCIs may be less effective in persons who cannot control gaze, such as severely disabled users (Brunner et al., 2010). This problem may be alleviated through different visual stimuli that do not require gaze shifting (Allison et al., 2008) or using a non-visual modality (Klobassa et al., 2009; Brouwer and van Erp, 2010). However, these solutions might reduce information transfer rate (Kübler et al., 2009).
3. P300 BCIs require a monitor or other external stimulation device to generate the flashes, tones, or other events that elicit P300s. Although subjects generally report that these events are not distracting or annoying, this could become a greater problem with long term use or different display parameters. On the other hand, research could focus further on displays that do not produce negative side effects. Also, even BCIs that do not rely on stimuli to generate events may still rely on stimuli for other aspects of system operation, such as an avatar's location in a virtual environment or feedback.
4. P300 BCIs are well suited to some tasks, and not others. P300 BCIs have been validated for tasks like spelling, smart home control, or internet browsing, which all entail direct selection. P300 BCIs may be less effective for other tasks. This problem might be reduced with new paradigms and tasks, or hybridizing with another BCI.

## Conclusions

As demonstrated, considerable progress has been made toward improving the P300 BCI paradigm, especially in the last few years. This is reasonable, since P300 BCIs have many appealing features including a very short training time and a high ITR. New avenues in P300 BCI research include paradigms for eliciting P300s, signal processing methods, applications, and hybrid BCIs. Future research should also investigate optimal electrode positions and the number of electrodes and to use source derivation and pre-processing algorithms to increase the SNR. Currently, mostly mono-polar recordings are used and the data are downsampled and bandpass filtered. These directions are creating new opportunities to make P300 BCIs faster, more accurate, easier to use, and better suited to the needs of individual users. Moreover, they are changing the face of P300 BCIs, since the canonical RC speller will be largely replaced with new types of spellers and wholly new applications such as Smart Home control or BP. Ongoing research efforts over the next several years will further develop P300 BCIs and related systems.

One issue that is rightly emerging as a major research direction is subjective reporting. Most BCI articles report only objective measures such as information transfer rate or accuracy. While these factors are important, they do not assess whether people enjoy using the BCI, ideally across the different conditions explored in the article. Thus, many articles may introduce new BCIs or paradigms that users dislike for some reason. Some recent studies have employed questionnaires to ask subjects whether using a P300 BCI is disturbing, annoying, tiring, and/or challenging (Guger et al., 2009, 2012; Muenssinger et al., 2010; Zickler et al., 2011; Jin et al., 2012). Subjects generally do not report significant problems with P300 BCIs. However, questionnaires and other means should still be employed to further explore subjective factors.

### Conflict of interest statement

The authors declare that the research was conducted in the absence of any commercial or financial relationships that could be construed as a potential conflict of interest.
